# Live Yeast and Yeast Cell Wall Supplements Enhance Immune Function and Performance in Food-Producing Livestock: A Review †,‡

**DOI:** 10.3390/microorganisms3030417

**Published:** 2015-08-07

**Authors:** Paul R. Broadway, Jeffery A. Carroll, Nicole C. Burdick Sanchez

**Affiliations:** United States Department of Agriculture, Agricultural Research Service, Livestock Issues Research Unit, Lubbock, TX 79403, USA; E-Mails: rand.broadway@ars.usda.gov (P.R.B.); nicole.sanchez@ars.usda.gov (N.C.B.S.)

**Keywords:** yeast, immunity, metabolism, livestock

## Abstract

More livestock producers are seeking natural alternatives to antibiotics and antimicrobials, and searching for supplements to enhance growth performance, and general animal health and well-being. Some of the compounds currently being utilized and studied are live yeast and yeast-based products derived from the strain *Saccharomyces cerevisiae*. These products have been reported to have positive effects both directly and indirectly on the immune system and its subsequent biomarkers, thereby mitigating negative effects associated with stress and disease. These yeast-based products have also been reported to simultaneously enhance growth and performance by enhancing dry matter intake (DMI) and average daily gain (ADG) perhaps through the establishment of a healthy gastrointestinal tract. These products may be especially useful in times of potential stress such as during birth, weaning, early lactation, and during the receiving period at the feedlot. Overall, yeast supplements appear to possess the ability to improve animal health and metabolism while decreasing morbidity, thereby enhancing profitability of these animals.

## 1. Introduction

In the current field of food production in America, farmers and ranchers are seeking natural technologies and compounds to improve animal health and performance. Furthermore, there is increasing pressure to accomplish these goals while minimizing or eliminating the use of antibiotics. A recent FDA guidance for industry recommended the removal of growth promotion claims from antibiotic labels [1] and most pharmaceutical companies have subsequently followed the guidelines. Fortunately, there are a plethora of natural alternatives to growth-promoting antibiotics that produce similar effects on performance and overall animal health and well-being [2,3]. One of these alternative products is live yeast and yeast cell wall products derived from *Saccharomyces cerevisiae*.

*Saccharomyces cerevisiae* is a species of yeast that has been used for centuries for a variety of processes including, but not limited to, brewing and bread-making. More recently, yeast and yeast cell wall components have been utilized as a supplement in the feeding of beef and dairy cattle [4,5], swine [6,7], lambs [8] and poultry [9]. Live yeast products and their derivatives (*i.e.*, yeast cell wall products) are currently utilized in food animal production for a variety of reasons encompassing performance enhancement and overall benefits to animal health and well-being [10].

Yeast products are often fed to livestock as either a live yeast (direct fed microbial; DFM), as yeast cell wall, or as a combination of the two. One of the major components of yeast and yeast cell wall products are polysaccharides such as α-d-glucan and β-d-glucan [11]. These polysaccharides not only interact directly with immune cells, but are also able to bind bacteria to prevent attachment and colonization of pathogens in the gastrointestinal tract [12]. In addition to pathogen mitigation [13], yeast cell wall components may possess antioxidant [14,15] and antitumor properties [16]. From an animal health and immunity perspective, yeast cell wall components from *Saccharomyces cerevisiae* have been reported to promote the release of cytokines from macrophages [17], and may be involved with modulation of immune cells in many species [18].

Yeast and yeast-based products not only alter immune function in animals, but they also possess the potential to enhance performance and alter metabolism. Thrune *et al.* [4] reported that dairy cows supplemented with *Saccharomyces cerevisiae* displayed less volatile fatty acid (VFA) concentrations in the rumen compared to control animals, and furthermore, yeast supplementation decreased duration of acidosis. Wohlt *et al.* [19] reported increases in dry matter intake (DMI) and milk yield in dairy cows in early lactation when supplemented with *Saccharomyces cerevisiae*. Increases in DMI and gain have also been reported in beef cattle supplemented with *Saccharomyces cerevisiae* during the receiving period [20]. Data from an *in vitro* study suggests that *Saccharomyces cerevisiae* supplements may also affect rumen pH and nutrient digestibility [21]. Yeast supplements affect live performance, although these supplements have also been reported to influence carcass performance and composition in growing lambs [8].

There are a plethora of positive effects associated with supplementation with *Saccharomyces cerevisiae* products. This review will briefly elucidate some of these effects with regard to immunity, metabolism, and performance. These natural supplements provide livestock producers with a “clean-label” feeding option that may possibly replace sub-therapeutic antibiotic supplementation while simultaneously mitigating the potential negative effects of morbidity on growth and performance. Importantly, the increases in performance observed with *Saccharomyces cerevisiae* supplementation coupled with decreased need of disease treatment may improve ultimate profitability.

## 2. Immunity and Health

Yeast and yeast cell wall products derived from *Saccharomyces cerevisiae* are immunomodulating compounds that interact directly and indirectly with both pathogens and components of the immune system [11,18]. Specifically, yeast and yeast derivatives may be involved in the synthesis and release stimulation of the pro-inflammatory cytokine TNF-α from macrophages [17] and are involved in the release of other cytokines such as IL-1, IL-2, and IL-6 [22,23]. The polysaccharide β-glucan is classified as a biological response modifier [24], and has been reported to increase the functionality of macrophages and neutrophils [25]. Increased T lymphocyte IFN-γ production has also been reported in swine supplemented with yeast products [26]. Other studies report alterations in the acute phase response (specifically IL-6 and cortisol) in feedlot heifers supplemented with *Saccharomyces cerevisiae* [27]. Additionally, Eicher *et al.* [2] reported increased TNF-α concentrations in multiple tissues in yeast supplemented pigs during a lipopolysaccharide challenge. Thus, yeast and yeast-based products may modulate and alter cytokine production and activation of the immune system in livestock possibly in part due to the priming effects of β-glucans on immune cells.

During the lifecycle of a food-producing animal, there may be various stressful events encountered by the animal that are evoked by pathogens and disease, environmental factors, handling stress, nutritional stress, relocation events, novel environments, and potentially other factors. During periods of stress, not only does activation of the hypothalamic-pituitary-adrenal (HPA) axis, and possibly the immune system, occur, but there also may be a loss in performance [28]. Currently, researchers are studying natural compounds which include products derived from *Saccharomyces cerevisiae* that may be supplemented to animals to mitigate some of these negative effects associated with stress. During heat stress, Burdick Sanchez *et al.* [29] reported that a yeast-based product was able to mitigate some of the negative effects associated with heat stress when accompanied by activation of the HPA axis in high-producing dairy cattle. In this study, the authors [29] reported decreased rectal temperatures in yeast-supplemented dairy cows during heat stress. Additionally, differences were observed in hematology when dairy cattle were subjected to heat stress along with activation of the HPA axis [29].

The receiving period is the time when young cattle first arrive at the feedlot and can be a stressful event that often results in increased susceptibility to disease and performance losses. Therefore, yeast supplements have been considered as a tool to mitigate some negative effects of this stress and to improve animal health and performance during the receiving period as well as during the entire feedlot period. In response to an acute inflammatory stress induced by lipopolysaccharide, Fink *et al.* [20] reported that yeast products improved the health of beef cattle during the receiving period (*i.e.*, the first 50 days in the feedlot) upon arrival to a feedlot. Duff and Galyean [30] also reported that improvements in health (*i.e.*, decreased morbidity) may be observed in yeast-supplemented cattle exposed to stress such as that associated with bovine respiratory disease or bovine viral diarrhea. Burdick Sanchez *et al.* [5] reported that yeast cell wall supplementation in receiving cattle enhanced the response to an acute immune challenge (*i.e.*, lipopolysaccharide), thus improving the probability of recovery and enhanced efficiency of incoming cattle. Specifically, the authors [5] reported that energy metabolism and nutrient utilization may have been enhanced in yeast supplemented heifers prior to an immune challenge characterized by decreased glucose, increased insulin, decreased non-esterified fatty acids (NEFAs) accompanied by alterations in BUN (blood urea nitrogen).

Other probiotics such as lactic acid producing bacteria have been utilized in livestock to enhance animal health and immunity, but few studies have evaluated combinations of probiotics from different sources. Emmanuel *et al.* [31] reported that when a bacterial probiotic was combined with a *Saccharomyces cerevisiae*, cattle produced an acute inflammatory response characterized by changes in the expression of lipopolysaccharide binding protein, haptoglobin, serum amyloid A, and other acute phase proteins. The authors [31] speculated this response may have been induced by pathogen lysis induced by the yeast supplement and immune priming.

Another stressful event in the life of an animal is immediately after birth. When colostrum deprived dairy calves challenged with *E. coli* were supplemented with *Saccharomyces cerevisiae*, the calves recovered from diarrheal symptoms faster that their non-supplemented cohorts [32]. A study by Magalhaes *et al.* [33] reported that incorporation of yeast supplements into the diets of dairy cattle enhanced performance while minimizing morbidity and mortality. Additionally, dairy calves supplemented with yeast products did not need to be treated as often, thus overall health and performance was improved in a manner in which profitability was substantially increased [33]. Furthermore, when pregnant cows were supplemented with mannan oligosaccharides, the antibody transfer and immunity of the offspring was enhanced [34].

In young swine, the immune system and cytokine production of the piglets was stimulated at an earlier age when supplemented with a yeast cell wall product [2]. White *et al.* [35] reported reduced colonization of coliforms throughout the gastrointestinal tract while minimally impacting the overall microflora. Additionally, supplementation with dried brewers yeast was reported to enhance immune biomarkers in weanling pigs [35]. While yeast supplements may directly enhance the health status of an animal, Shen *et al.* [36] reported that supplementation of a *Saccharomyces cerevisiae* to gestating sows can influence the health of subsequent offspring by altering hematology, specifically neutrophils. Additionally, researchers have reported positive effects on the immune system in weaned piglets when fed mannan-oligosaccharides derived from yeast fermentation products [37].

The mechanisms of action by which probiotics such as yeast and yeast products influence immunity and health have been investigated [35,36,37,38,39]; however, they have not been fully elucidated at this time. One of the mechanisms by which *Saccharomyces cerevisiae* influences health and immunity is by alterations in the microbiome of the gastrointestinal tract of animals [38]. Mullins *et al.* [39] reported a decrease in *Eubacterium ruminatium* corresponding to increased DMI. This bacterium is a common and abundant cellulytic organism in the rumen [40]. *Saccharomyces* supplemented animals additionally exhibited differences in *Ruminococcus albus* and fungal populations [39]. Studies have reported that development of a healthy intestinal flora can enhance nutrient uptake while simultaneously improving immune function [41,42,43]. Some strains of *Saccharomyces cerevisiae* have been reported to promote fibrolytic bacteria in the gastrointestinal tract of ruminants and may improve gut health during transition to a solid diet [44]. Yeast cell wall polysaccharides may also inhibit the binding and colonization of bacterial pathogens in the gastrointestinal tract [45] which can mitigate subsequent infections. More recent data suggests that direct binding occurs between yeast cell wall components as well as live yeast probiotics derived from *Saccharomyces cerevisiae* and pathogenic bacteria such as *E. coli*, *Salmonella*, and *Listeria* [46]. Immune modulating compounds commonly found in yeast and yeast products also possess the ability to inhibit the onset of disease caused by protozoan and viruses [47,48]. Some research has specifically investigated the effects of yeast cell wall components on leukocytes, and researchers noted increases in pro-inflammatory cytokines, oxidative bursts, and chemotaxis [49,50,51,52]. While enhancement of immune biomarker expression is helpful during the occurrence of an immunological insult, the production of these compounds increases energy requirements and may not be beneficial to performance in a healthy animal.

## 3. Performance and Metabolism

Studies involving yeast products have reported increased weight gain and a general enhancement of animal health and well-being [53,54,55]. Studies have also reported that *Saccharomyces cerevisiae* supplemented to ruminants can affect DMI, rumen pH and overall nutrient digestibility [21,56]. However, the modes of action of yeast products that directly or indirectly impact these metabolic and performance changes have not been fully elucidated.

While yeast and yeast products may enhance performance via mechanisms that enhance animal health (specifically related to bacterial colonization in the gastrointestinal tract), these products may also increase nutrient digestibility [57,58] as reported in dairy cows. Kumar *et al.* [59] observed elevated bacterial populations in the rumen accompanied by altered fatty acid production. Specifically, concentrations of cellulolytic and amylolytic bacteria were increased in the rumen thus increasing the acetate:propionate ratio in *Saccharomyces*-supplemented animals [59]. Wohlt *et al.* [19] reported increases in milk yield and protein digestibility in early lactating dairy cows. Another study also reported increases in milk yield and DMI while also allowing the dairy cows to reach peak milk production sooner [56]. While most literature suggests performance enhancements, a study by Hadjipanayiotou *et al.* [60] reported yeast supplements had no impact on performance in dairy ewes.

As reported before, the positive effects of yeast and yeast products have been observed in dairy cattle, they have also been reported in beef cattle and swine. Finck *et al.* [20] reported increases in DMI in cattle during the receiving period at the feedlot when cattle were supplemented with yeast products. Other studies utilizing supplementation with yeast products have also reported increases in DMI and average daily gain (ADG) of feeder cattle [61]. Eicher *et al.* [2] reported an increase in ADG and body weight BW when pigs were supplemented with a yeast cell wall product while undergoing an immune challenge. Yeast and yeast cell wall products have been reported in other studies to enhance live performance [62,63] and to alter intestinal morphology in swine [64]. Increases in ADG have been reported in weaned piglets supplemented with yeast products [65], and feed:gain has been reported to decrease when weaned piglets were fed yeast products [66]. However, when yeast cell wall extract was supplemented to weaned piglets, Sauerwein and authors [67] reported no difference in performance and growth parameters.

In addition to the improvement of animal health and reduction of risk of disease, yeast products may also enhance performance and metabolism during stressful events. In a review, Duff and Galyean [30] reported increases in cattle performance in yeast-supplemented cattle exposed to bovine respiratory disease. In response to a lipopolysaccharide challenge, yeast supplementation was reported to improve energy metabolism thereby inhibiting lipolysis and protein catabolism [5] which corresponded to an 8–11 kg increase in BW and increases in ADG averaging 0.1 kg. In a study of colostrum-deprived newborn calves supplemented with *Saccharomyces cerevisiae* in replacement of milk, supplemented calves displayed increased intake, weight gain, and plasma glucose concentrations [32].

*Saccharomyces* yeast supplementation during gestation may influence characteristics of offspring. Shen *et al.* [36] reported when sows were supplemented with a *Saccharomyces cerevisiae* product, their progeny exhibited increased weaning weight by approximately 1.0 kg and increased ADG. Additionally, yeast-supplemented sows exhibited enhanced maternal protein utilization by reducing plasma urea nitrogen [36]. Yeast products not only alter live performance, but also influence differences in carcass performance and meat quality. A study by Tripathi and Karim [8] reported increases in live weight gain without altering carcass quality; however, yeast products were able to influence carcass composition in multiple primal cuts.

## 4. Conclusions

Overall, as can be gleamed from the literature, yeast-based products have been reported to enhance immunity and health during stressful events by acting as immunomodulators and biological response modifiers, thereby mitigating some of the negative effects associated with pathogenesis and morbidity. In part by enhancing general gut health and modifying the composition of the microbiome, these supplements also influence the metabolism of food-producing livestock. These metabolic shifts have been reported to enhance performance and growth without negatively effecting milk production or carcass characteristics. These compounds represent a class of natural supplements that may partially replace the need for antibiotics and treatments due to illness. The combination of these factors has the ability to enhance animal health and well-being which may ultimately impact profitability.
